# Epidemiology of Primary Multidrug-Resistant Tuberculosis, Vladimir Region, Russia

**DOI:** 10.3201/eid2111.150813

**Published:** 2015-11

**Authors:** Julia V. Ershova, Grigory V. Volchenkov, Dorothy A. Kaminski, Tatiana R. Somova, Tatiana A. Kuznetsova, Natalia V. Kaunetis, J. Peter Cegielski, Ekaterina V. Kurbatova

**Affiliations:** Centers for Disease Control and Prevention, Atlanta, Georgia, USA (J.V. Ershova, D.A. Kaminski, J.P. Cegielski, E.V. Kurbatova);; Vladimir Oblast TB Dispensary, Vladimir, Russia (G.V. Volchenkov, T.R. Somova, T.A. Kuznetsova, N.V. Kaunetis)

**Keywords:** multidrug resistance, primary multidrug-resistant tuberculosis, MDR TB, tuberculosis and other mycobacteria, transmission, bacteria, Russia, epidemiology, Mycobacterium tuberculosis, antimicrobial resistance

## Abstract

We studied the epidemiology of drug-resistant tuberculosis (TB) in Vladimir Region, Russia, in 2012. Most cases of multidrug-resistant TB (MDR TB) were caused by transmission of drug-resistant strains, and >33% were in patients referred for testing after mass radiographic screening. Early diagnosis of drug resistance is essential for preventing transmission of MDR TB.

Drug-resistant tuberculosis (TB) is a public health problem worldwide (*1*). Compared with drug-susceptible TB, multidrug-resistant TB (MDR TB; i.e., TB with resistance to at least isoniazid and rifampin) requires longer, more expensive treatment and is less likely to be cured (*2*,*3*). Russia has the third highest burden worldwide of MDR TB; ≈41,000 pulmonary cases were notified in 2013 (*1*). The World Health Organization estimated that 19% of new TB cases notified in Russia in 2013 were primary MDR TB (*1*); however, drug-susceptibility testing (DST) coverage varies across the country (*1*,*4*). Prevalence of primary MDR TB varied from 5.4% to 28.3% among the 12 regions in Russia that reported MDR TB data in 2010 (*5*). Understanding the burden of drug-resistant TB and the factors associated with its transmission may help determine who is at risk for infection and develop measures for preventing transmission. We describe the epidemiology of primary MDR TB in Vladimir Region, Russia.

## The Study

During February 1–December 31, 2012, we performed a secondary analysis of data collected for a study conducted at the Vladimir Regional TB Dispensary (hereafter referred to as the dispensary), which is located 190 km east of Moscow in Vladimir Region (Figure). The dispensary, a referral center for TB patients in Vladimir Region, serves ≈25% of all TB patients in the region and is the only facility in the region that performs DST.

**Figure:**
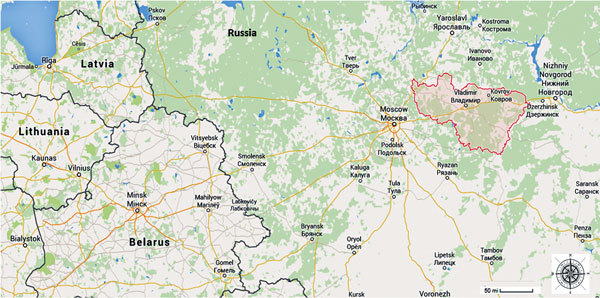
Vladimir Region in Russia (red shading). Map source: Google Maps, Vladimir Oblast, Russia.

All adults referred to the dispensary for suspected TB during the study period were eligible for study enrollment. Study participants underwent clinical examination, chest radiography, and HIV testing. In addition, clinical samples were obtained for culture and DST, which was performed by using Lowenstein-Jensen media and Bactec Mycobacteria Growth Indicator Tube 960 (MGIT; Becton, Dickinson and Company, Franklin Lakes, NJ, USA). In August 2012, the Xpert MTB/RIF (Xpert; Cepheid, Sunnyvale, CA, USA) diagnostic test became available for routine patient care at the dispensary. We used rifampin resistance, which we defined as resistance detected by Lowenstein-Jensen media, MGIT DST, or Xpert, as a marker for MDR TB (*6*). Rifampin susceptibility was defined as susceptibility by all 3 testing methods or by any 1 of the 3 methods when results from all 3 methods were not available. Primary MDR TB was defined as the presence of rifampin resistance in new TB cases.

We described the epidemiology of new TB and MDR TB cases that were diagnosed in the dispensary during the study period. To identify the predictors of primary MDR TB, we compared the characteristics of MDR TB and non–MDR TB case-patients by using bivariate log-binomial regression.

The study was approved by ethics committees at the Centers for Disease Control and Prevention and the Central Tuberculosis Research Institute. All patients provided written informed consent.

During the study period, samples from 402 patients with presumptive TB were tested bacteriologically in the dispensary. Among these samples, 229 (56.9%) were *Mycobaterium tuberculosis*–positive by culture or Xpert. Of these 229 samples, 225 (98.3%; 191 from new TB patients, 34 from previously treated TB patients) were tested for rifampin resistance; 60 (26.7%) showed MDR TB. Forty-four (23.0%) of the 191 samples from new TB patients and 16 (47.1%) of the 34 samples from previously treated TB patients showed MDR TB (prevalence ratio 1.45, p = 0.004). The proportion of primary MDR TB cases among all MDR TB cases was 73.3% (44/60).

The median age of the 191 new TB patients at the time of diagnosis was 39 years; 146 (74.4%) were men (Table). Most new TB patients had pulmonary disease (96.3%, 183/191) and positive sputum smear results (73.7%, 140/191); 18 (9.6%) were HIV-positive. Lowenstein-Jensen media or MGIT DST results were available for 91.6% (175/191) of new TB case-patients; isoniazid monoresistance was detected in 16.0% (28/175). No rifampin monoresistance was found.

**Table:** Association between sociodemographic and clinical characteristics of persons with newly diagnosed cases of non–MDR TB and MDR TB, Vladimir Region, Russia, 2012*

Characteristic	Total, n = 191	MDR TB, n = 44	Non–MDR TB, n = 147	Prevalence ratio	95% CI	p value
Sex						
Male	146 (74.4)	34 (77.3)	112 (76.2)	1.05	0.56–1.95	0.88
Female	45 (23.6)	10 (22.7)	35 (23.8)	Referent		
Median age, y (interquartile range)†	39 (30–49)	36 (30–46)	40 (30–50)			0.34‡
HIV†						
Positive	18 (9.6)	7 (16.3)	11 (7.6)	1.8	0.96−3.51	0.09
Negative	170 (90.4)	36 (83.7)	134 (92.4)	Referent		
Health care worker						
Yes	2 (1.1)	1 (2.3)	1 (0.7)	2.2	0.54–9.01	0.27
No	189 (98.9)	43 (97.7)	146 (99.3)	Referent		
Homelessness						
Yes	11(5.8)	4 (9.1)	7 (4.8)	1.64	0.72−3.75	0.24
No	180 (94.2)	40 (90.9)	140 (95.2)	Referent		
Employment						
Employed	42 (22.0)	10 (22.7)	32 (21.8)	Referent		
Unemployed	114 (59.7)	25 (56.8)	89 (60.5)	0.92	0.48–1.75	0.80
Not working§	35 (18.3)	9 (20.5)	26 (17.7)	1.08	0.49–2.35	0.85
History of imprisonment ¶						
Yes	51(26.8)	10 (22.7)	41 (28.1)	0.8	0.43–1.50	0.49
No	139 (73.2)	34 (77.3)	105 (71.9)	Referent		
Alcohol abuse						
Yes	35 (18.3)	9 (20.5)	26 (17.8)	1.15	0.61–2.16	0.67
No	156 (81.7)	35 (79.5)	121 (82.3)	Referent		
Illicit drugs use#						
Yes	12 (6.3)	3 (7.0)	9 (6.2)	1.11	0.40–3.06	0.85
No	177 (93.7)	40 (3.05)	137 (93.8)	Referent		
Diabetes¶						
Yes	13 (6.8)	3 (7.0)	10(6.8)	1.02	0.36–2.86	0.97
No	177 (93.2)	40 (93.0)	137 (93.2)	Referent		
Contact with TB patient						
Yes	55 (28.8)	16 (36.4)	39 (26.5)	1.45	0.85–2.49	0.17
No	130 (68.1)	26 (59.1)	104 (70.7)	Referent		
Unknown	6 (3.1)	2 (4.5)	4 (2.8)	–		
Cavities on radiograph¶						
Yes	88 (46.3)	24 (54.5)	64 (43.8)	1.39	0.83–2.34	0.21
No	102 (53.7)	20 (45.5)	82 (56.2)	Referent		
Sputum microscopy¶						
Positive	140 (73.7)	39 (88.6)	101(69.2)	2.78	1.16–6.67	0.02
Negative	50 (26.3)	5 (11.4)	45 (30.82)	Referent		
Site of disease¶						
Any pulmonary	189 (99.5)	44 (100)	145 (99.3)			0.59
Extrapulmonary only	1 (0.5)	0	1 (0.7)	Referent		
Symptoms						
Yes	122 (63.9)	27 (61.4)	95 (64.6)	0.9	0.53–1.53	0.69
No	69 (36.1)	17 (38.6)	52 (35.4)	Referent		
Reasons for TB screening						
Symptomatic	110 (57.6)	27 (61.4)	83 (56.4)	1.1	0.62–1.79	0.85
Abnormal finding on radiograph	6 (3.1)	0	6 (4.1)	–		
Contact with TB patient	2 (1.1)	0	2 (1.4)	–		
Routine screening	73 (38.2)	17 (38.6)	56 (38.1)	Referent		

*Values are no. (%) patients except as indicated. Cases were diagnosed at the Vladimir Regional TB Dispensary, Vladimir Region, Russia. MDR TB, multidrug-resistant TB. –, not applicable. †Age and HIV status missing for 3 patients. ‡Wilcoxon rank sum test. §Reasons for not working: retired, student, housewife, disabled. ¶Information was missing for 1 patient. #Drug use data were missing for 2 patients.

Among 44 study participants with primary MDR TB, 17 (38.6%) had been referred to the dispensary for TB examination after routine mass radiographic screening in the primary health care setting (Table); 15 (88.2%) of these 17 patients were asymptomatic at the time of diagnosis. Of the 44 patents with primary MDR TB, 27 (61.4%) reported TB symptoms at diagnosis, and 16 (35%) had known contact with someone with TB. All patients with primary MDR TB had pulmonary TB disease. *M. tuberculosis* isolates from 4 (9.1%) of the 44 patients with primary MDR TB were also resistant to at least 1 second-line injectable drug and a fluoroquinolone; therefore, these 4 patients had primary extensively drug-resistant TB.

Among newly diagnosed TB case-patients, a positive sputum smear was the only factor significantly associated with MDR TB (prevalence ratio 2.8, 95% CI 1.2–6.7) (Table). The prevalence ratio for the association of HIV positivity with primary MDR TB suggested an increased, but not statistically significant, risk (prevalence ratio 1.8, 95% CI 0.96–3.50).

## Conclusions

Primary MDR TB was prevalent in Vladimir Region during 2012, matching the World Health Organization prevalence estimate for Russia (*1*). Most persons in this study who received a diagnosis of MDR TB (73.3%, 44/60) had not previously received TB treatment, a finding that indicates ongoing transmission of drug-resistant *M. tuberculosis* in the community. This percentage is near the upper end of the range of primary MDR TB prevalence (31%–82%) reported in a review of data for 30 countries (*7*). In settings with a high prevalence of primary MDR TB, not just previously treated TB patients but new TB patients as well should be included in MDR TB case-finding strategies. Russia does not have full DST coverage (*1*); however, in Vladimir Region, all bacteriologically confirmed TB case-patients, including new case-patients, must undergo MGIT DST.

Our findings show that 38.6% of primary MDR TB cases were detected by routine radiographic screening, and most of these patients lacked symptoms at diagnosis. In Russia, mass chest radiographic screening is conducted every 1–2 years. The screening is part of the mandatory occupational health screenings for persons in some high-risk occupations and for patients with certain medical conditions, and it is also included in routine prophylactic health screenings. Active TB case finding in combination with early detection of drug resistance, supported by universally available DST and Xpert diagnostics, may substantially reduce transmission of MDR TB in the community.

A positive sputum smear was the only significant predictor of MDR TB among patients with primary TB. MDR TB was more common among HIV-infected than non–HIV-infected new TB patients; this finding was similar to those in cross-sectional studies conducted in several Eastern European countries, although this association was not statistically significant in our study (*5*,*8*,*9*). A high level (16%) of isoniazid monoresistance detected among previously untreated TB case-patients is concordant with findings from other regions in Russia (*10*).

Our study had several limitations. The small sample size limited statistical power. Xpert was introduced halfway through the study. Nonavailability of conventional DST for some patients limited our ability to analyze resistance pattern among all enrolled patients. There was a possibility of misclassification of treatment history. We did not include patients from the penitentiary sector or from other diagnostic facilities in the region; thus generalizability of our results may be limited. The lack of networking data and molecular epidemiology may also present a limitation for the study conclusions.

Despite these limitations, our results show that primary MDR TB was common in the overall burden of MDR TB in Vladimir Region. Active case finding and expansion of DST or Xpert testing to new TB patients are key measures for achieving universal access to MDR TB diagnosis and treatment and for preventing the spread of drug-resistant TB in the community.
